# Vascular Effects of Dietary Advanced Glycation End Products

**DOI:** 10.1155/2015/836498

**Published:** 2015-05-18

**Authors:** Alin Stirban, Diethelm Tschöpe

**Affiliations:** ^1^Profil Institute for Metabolic Research, Hellersbergstraße 9, 41460 Neuss, Germany; ^2^Diabetes Clinic, Heart and Diabetes Center NRW, Ruhr University Bochum, 32545 Bad Oeynhausen, Germany

## Abstract

Evidence has accumulated lately demonstrating that advanced glycation end products (AGEs) play an important role in the development of diabetic and cardiovascular complications as well as the development of other chronic diseases. AGEs originating from diet have a significant contribution to the AGEs body pool and therefore dietary interventions aiming at reducing AGEs load are believed to exert health promoting effects. This review summarizes the evidence from clinical studies regarding effects of dietary AGEs on the vascular system, highlighting also the different aspects of vascular tests. It also advocates an extension of dietary recommendations towards the promotion of cooking methods that reduce dietary AGEs in consumed foods.

## 1. Introduction

Cardiovascular disease (CVD) belongs to the leading causes of increased mortality and morbidity in diabetes and many attempts have been made to lower its incidence [1, 2]. Endothelial dysfunction (ED) represents an early, reversible stage of atherosclerosis and is exacerbated in subjects with diabetes mellitus [3]. Several studies suggested, for example, the assessment of endothelial function by ultrasound (e.g., the measurement of flow-mediated dilatation, FMD) to be a prognostic factor for cardiovascular (CV) events [4–6]. Indeed, FMD improves with therapies that decrease cardiovascular risk in people with diabetes, suggesting that restoration of endothelial function might promote CV health, while its impairment promotes atherosclerosis [7, 8].

## 2. Measurement of Endothelial Function

The measurement of endothelial function has become an important tool for both clinicians and researcher for at least 3 reasons: it allows the identification of patients at risk; it can be used to measure positive vascular effects of different interventions (e.g., medicaments); and it enables the quantification of detrimental vascular effects, for example, during the postprandial state.

Early identification of patients at high risk is clinically important enabling the implementation of more intensive prevention strategies. Therefore, several methods have been developed and proved their efficacy in predicting CV risk, with the ultrasound measurement of FMD of the brachial artery being one of the widest used [9, 10]. Indeed, several studies have shown that the measurement of FMD predicts cardiovascular risk and improves risk stratification especially in patients with known CVD [11–13]. FMD has the advantage that it is noninvasive and has a good repeatability (when conditions are well standardized), but it requires special equipment and highly skilled investigators. Moreover, this technique investigates the endothelial function of conductance vessels. Endothelial function of conductance vessels underlies a different regulation than that of the microcirculation and a growing body of evidence suggests that the microcirculation might be the initial site where endothelial damage occurs [14]. Therefore, techniques investigating microvascular function have been developed too. They add to the early prediction of CV risk in the general population and the risk for the development of diabetes complications in subjects with diabetes mellitus. At least, three comprehensive reviews on available methods for the evaluation of peripheral neurovascular function have been made available lately by Vinik et al. [15], Cracowski et al. [16], and Stirban [17]. One of the most used methods assesses with a single-point laser-Doppler the skin reactive hyperemia following transient ischemia of the forearm [18]. The method has several advantages: it is investigator independent and it can be performed in parallel to the FMD, thus enabling the assessment of both macrovascular and microvascular endothelial functions.

Beyond the above-mentioned functional tests of micro- and macrocirculation, biomarkers of endothelial dysfunction are available like E-selectin, intercellular adhesion molecule-1 (ICAM-1), and vascular cell adhesion molecule-1 (VCAM-1) [19].

## 3. Postprandial State and Postprandial Endothelial Dysfunction

Zilversmit [20] called already in 1979 the atherogenesis to be a “postprandial phenomenon.” Indeed, there is compelling data linking postprandial or postload hyperglycemia [21] and hypertriglyceridemia [22] to cardiovascular events suggesting that the postprandial state plays an important role in the development of CVD [23, 24]. The pathogenic importance of the postprandial state becomes even more understandable if we consider that it covers around 2/3 of our daytime.

The mechanisms that link postprandial dysmetabolism to CVD might be the exacerbation of oxidative stress and the occurrence of endothelial dysfunction [25–27].

At least 4 factors deteriorate postprandial endothelial function: hyperglycemia, hypertriglyceridemia, hyperinsulinemia, and food toxins like the so-called advanced glycation end products (AGEs). Postprandial hyperglycemia and hypertriglyceridemia have not only independent but also cumulative effects on postprandial endothelial dysfunction both in healthy subjects and in diabetic patients [28]. Campia and coworkers demonstrated that acute hyperinsulinemia impairs conduit vessel endothelial function independent of insulin sensitivity and lipid profile concluding that hyperinsulinemia may trigger ED and promote atherosclerosis too [29]. Several study groups, including ours, have extensively investigated the effects of food AGEs in humans.

## 4. AGEs: Definition, Pathogenic Effects, and Sources

AGEs are a heterogeneous group of compounds formed by the nonenzymatic glycation of proteins, lipids, or nucleic acids [30, 31] within the so-called “Maillard reaction.” The reaction was named in honor of the French scientist Louis Camille Maillard (1878–1936). It consists of several steps; the first, reversible step takes place between the carbonyl group of a reducing sugar such as glucose and an aminoterminal group of a protein, lipid, or nucleic acid generating a so-called “Schiff base.” By structural irreversible rearrangements, more stable ketoamines are formed, called Amadori products (such as the HbA1_c_) [32]. The Amadori products undergo further structural changes through a series of reactions such as oxidation, dehydration, and degradation to finally yield highly stable AGE compounds [32, 33].

We have recently reviewed the pathogenic mechanisms of AGEs on the vascular system [34]. Briefly, some of the mechanisms are related to inflammation and oxidative stress [35], increased glycation of low-density and high-density lipoproteins (LDL and HDL) [36], activation of the proinflammatory inducible nitric oxide- (NO-) synthase (iNOS) [37], and inhibition of NO availability [38]. Further mechanisms comprise the increased production of cytokines, for example, insulin-like growth factor-1 (IGF-1) or the platelet derived growth factor (PDGF), which modify the migration of monocytes and macrophages as well as the proliferation of vascular smooth muscle cells (VSMC) [39, 40]. Overall, AGEs exert their deleterious effects by receptor-dependent mechanisms and receptor-independent mechanisms. Receptor-independent effects comprise glycation of proteins and lipoproteins (thus altering their normal function [41]), glycation of LDL particles on the apolipoprotein B (ApoB) and phospholipid components [42, 43], glycation of matrix proteins such as collagen VI, laminin, and vitronectin [44], and so forth. AGEs receptors are present on the surface of different cell types such as macrophages, adipocytes, endothelial cells, and vascular smooth muscle cells (VSMC) and several types of receptors including scavenger receptors (macrophage scavenger receptor-AI, macrophage scavenger receptor-AII, CD68, and CD36), RAGE, AGE-R1, AGE-R2, and AGE-R3 have been described, [45, 46]. Although scavenger receptors are responsible for the removal of AGEs, RAGE likely mediates most biological effects of AGEs [47]. The AGEs-RAGE interaction triggers oxidative stress, inflammation, and apoptosis [48–50].

AGEs can be formed within the organism (endogenous source) or can originate from exogenous sources [34]. Although AGEs are better known as by-products of hyperglycemia, they also form within food during heat-enhanced cooking [51]. Evidence has accumulated that dietary AGEs are partially absorbed [52, 53] and either retained in the body or excreted in the urine [53–55]. These dietary AGEs represent an important source for circulating AGEs under in vivo conditions [56–58]. Moreover, smoking also serves as an additional exogenous source of AGEs [59].

The amount of AGEs in food is dependent on the nutrients used, but the AGEs concentration can be greatly influenced by the cooking method. The AGEs generation during cooking increases with temperature, decreasing moisture, cooking time, and increased pH [51]. An excellent article by Uribarri and colleagues provides information on the AGEs amount in several hundreds of meals; this database provides a valuable instrument for estimating food AGEs and contains recommendations of how to reduce dietary AGEs intake [51].

## 5. In Vivo Effects of Food AGEs

We have recently reviewed [34] the vascular effects of food AGEs in animal models, highlighting that higher circulating concentrations of AGEs, particularly food-derived AGEs, can induce cross-linking of arterial wall connective tissue protein [60], aortic atherosclerotic lesions [61], and neointimal formation after arterial injury [62], increase vascular permeability, and markedly impair vascular vasodilatory response [63]. Moreover, dietary AGEs restriction improves insulin sensitivity, prevents from the development of diabetic nephropathy, and increases life span [64, 65].

Chilelli et al. [66] reviewed the importance of AGEs for the development of microvascular complications in diabetes advocating that their prevention and treatment must focus not only on early glycemic control, but also on reducing oxidative stress, and especially the dietary intake of exogenous AGEs.

Indeed, several studies have investigated the effects of food AGEs in humans. These studies will be briefly presented and discussed especially from the perspective of vascular effects. For other aspects of the effects of dietary AGEs in humans, two excellent reviews on AGEs in food and their effects on health have been recently made available by Poulsen et al. [67] and Kellow and Savige [68].

Most of the studies investigating effects of dietary AGEs on health dealt with at least 3 important questions. First, at what amount can a dietary modulation of AGEs influence circulating AGEs concentration in populations with different AGEs load? Second, can dietary AGEs modulation influence endothelial function? Third, are these effects due to AGEs or rather due to other dietary toxins generated during cooking?

These questions were addressed mainly in 3 populations: healthy subjects (having a low endogenous AGEs production and an unaltered renal AGEs excretion), subjects with diabetes mellitus (high endogenous AGEs production), and subjects with renal failure (exacerbated endogenous AGEs production and reduced renal excretion).

## 6. Modulation of Circulating AGEs by Changing the Dietary AGEs Load

From clinical point of view, it makes sense to reduce dietary AGEs only if this has a substantial impact on circulating AGEs. Several kinetic studies have suggested that approximately 10–30% of dietary AGEs are absorbed and around one-third of ingested AGEs are excreted into the urine and feces [69]. An important contributor to circulating AGEs seems to be also the capacity of the body to eliminate AGEs [55, 70].

An acute intervention demonstrated in patients with type 2 diabetes mellitus that the ingestion of a single meal with a high AGEs (H-AGEs) content increased carboxymethyllysine (CML) by 15.6%^∗^ and methylglyoxal (MG) by 20.7%^∗‡^ compared to fasting, while following a meal with a low AGEs (L-AGEs) content CML decreased by 5.4% and MG decreased by 10% (^∗^ *P* < 0.05 versus low AGEs, ^‡^ *P* < 0.05 versus fasting) [18]. In healthy subjects, the intake of a beverage containing AGEs increased serum AGEs by 29% [71].

Chronic interventions influence circulating AGEs at an even higher degree. In nondiabetic, renal failure patients on peritoneal dialysis, 4 weeks of low dietary L-AGEs intake decreased serum CML by 34%^∗^ and serum MG by 35%^∗^, while a H-AGEs intake of similar length increased serum CML by 29%^∗^ and serum MG by 26%^∗^ (^∗^ *P* < 0.05 versus baseline) [57]. In patients with diabetes, following a dietary intervention over 6 weeks, serum AGEs were increased by 28.2% on H-AGEs (*P* = 0.06 versus baseline) and reduced by 40% on L-AGEs (*P* < 0.02 versus baseline) [56].

In healthy subjects exposed in a cross-over manner to a dietary intervention of 1 month each, plasma CML was significantly higher (7%, *P* = 0.002) after the H-AGEs diet than after the L-AGEs diet [72]. In another study, in healthy adults, a 6-week L-AGEs diet reduced serum CML from 763 ± 24 to 679 ± 29 ng/mL (−11%, *P* = 0.03) and urine CML from 1.37 ± 1.47 to 0.77 ± 2.01 *μ*g/mL creatinine (−43%, *P* = 0.02) [73].

Overall, it seems that dietary interventions significantly influence circulating AGEs. It is important to note that the absorption of different AGEs varies greatly and also the fact that usually for the assessment of the AGEs load measurements of CML, MG, or pentosidine are used. It is still a matter of debate whether these AGEs are representative for the dietary AGEs class and whether they can be mainly made responsible for the deleterious effects of AGEs.

## 7. Can Dietary AGEs Modulation Influence Endothelial Function?

In a proof of principle study [18], we tested the hypothesis that a single “real-life” H-AGEs-meal acutely induces more pronounced vascular dysfunction than does a low-AGEs (L-AGEs) meal matched for caloric as well as micronutrient and macronutrient content. We performed a randomized, crossover study, investigating in 20 in-patients with type 2 diabetes the effects of L-AGEs and H-AGEs meal on macrovascular (assessed by flow-mediated dilatation (FMD)) and microvascular (assessed by laser-Doppler flowmetry) function, serum markers of endothelial dysfunction (E-selectin, intracellular adhesion molecule 1, and vascular cell adhesion molecule 1), oxidative stress, and serum AGEs. The meals had identical ingredients but different AGE amounts (15.100 compared with 2.750 kU AGE for the H-AGEs and L-AGEs meals, resp.), which were obtained by varying the cooking temperature and time. Vascular measurements were performed at baseline and 2, 4, and 6 h after each meal. Following the H-AGEs meal, FMD decreased by 36.2%, from 5.77 ± 0.65% (baseline) to 3.93 ± 0.48 (2 h), 3.70 ± 0.42 (4 h), and 4.42 ± 0.54% (6 h) (*P* < 0.01 for all compared with baseline). After the L-AGEs meal, FMD decreased by 20.9%, from 6.04 ± 0.68% (baseline) to 4.75 ± 0.48% (2 h), 4.69 ± 0.51% (4 h), and 5.62 ± 0.63% (6 h), respectively (*P* < 0.01 for all compared with baseline; *P* < 0.001 for all compared with the HAGEs meal). This impairment of macrovascular function after the HAGE meal was paralleled by an impairment of microvascular function (−67.2%) and increased concentrations of serum AGE and markers of endothelial dysfunction and oxidative stress. Following both meals, glucose, triglycerides, and insulin excursions were comparable. We concluded that, in patients with T2DM, HAGEs meal induces a more pronounced acute impairment of vascular function than does an otherwise identical LAGEs meal. Therefore, chemical modifications of food by means of cooking play a major role in influencing the extent of postprandial vascular dysfunction.

In contrast, in 19 healthy individuals completing a cross-over trial, Poulsen et al. showed that a single high-AGEs meal compared to a low-AGEs meal did not show effects on appetite and markers of inflammation or endothelial activation but affected postprandial ghrelin, oxidative stress (urinary F2-isoprostanes), and glucose responses [74]. FMD and functional microvascular tests were not applied in this study; therefore, the comparison to our study is limited.

Several studies investigated the effects of chronic dietary AGEs modulation on endothelial function in patients with type 2 diabetes mellitus.

In one study performed in 24 diabetic subjects, Vlassara et al. [56] investigated the effects of two equivalent diets, one regular (H-AGEs) and the other with 5-fold lower AGEs (L-AGEs) content on inflammatory mediators and markers of ED. Eleven of the subjects participated in a 2-week crossover and 13 subjects in a 6-week parallel study. The authors demonstrated that, after 2 weeks on H-AGEs, serum AGEs increased by 64.5% (*P* = 0.02) and on L-AGEs decreased by 30% (*P* = 0.02) and serum vascular adhesion molecule-1 was 1,108 ± 429 and 698 ± 347 ngml (*P* = 0.01) on H- and L-AGEs, respectively. After 6 weeks, vascular adhesion molecule-1 declined by 20% on L-AGEs (*P* < 0.01) and increased by 4% on H-AGEs. This study shows that, lowering dietary AGEs, a significant reduction in circulating AGEs levels can be achieved, along with a decrease in soluble factors that mirror ED.

In another study, Cai and coworkers [75] demonstrated that, in 24 diabetic subjects randomized to either a standard diet (H-AGEs) or a diet 5-fold lower in AGEs (L-AGEs) for 6 weeks, LDL pooled from patients on H-AGEs diet was more glycated than LDL pooled from the L-AGEs diet group (192 versus 92 AGE U/mg apolipoprotein B) and more oxidized (5.7 versus 1.5 nmol malondialdehyde/mg lipoprotein). They noted that the LDL pooled from patients on H-AGEs added to human endothelial cells promoted marked ERK1/2 phosphorylation (pERK1/2) (5.5- to 10-fold of control) and stimulated NF-kappaB activity compared to the LDL pooled from patients on L-AGEs. Since glycated and oxidized LDL might promote atherosclerosis, this study shows that dietary AGEs might have atherogenic effects by enhancing LDL-induced vascular toxicity via redox-sensitive mitogen-activated protein kinase activation, an effect that can be reduced by dietary AGEs restriction.

Peppa et al. [76] demonstrated in a group of 18 patients with chronic renal failure randomly assigned to a 4-week diet with either L-AGEs or H-AGEs that dietary AGEs modulation resulted in a significant decrease in levels of serum AGEs, CRP, and PAI-1 in the L-AGEs group (approximately 35%, 44%, and 17%, resp.; *P* < 0.03), whereas only serum AGEs levels increased significantly in the H-AGEs group. VCAM-1 and TNF-alpha levels, although similar at baseline, became significantly lower in patients on L-AGEs compared with H-AGEs diet (*P* < 0.05) at the end of the study.

Patients with diabetes mellitus and/or renal failure show increased AGEs production and reduced AGEs renal clearance. But healthy subjects have presumably intact protective mechanisms that might compensate an increased dietary AGEs load. Therefore, the question arises whether dietary AGEs restriction has also quantifiable effects in healthy subjects.

The study by Birlouez-Aragon dealt with this question and investigated the effects of dietary AGEs in healthy subjects [72]. The study performed in 62 volunteers was a randomized, crossover, diet-controlled intervention trial with the duration of 4 weeks, designed to compare the potential metabolic effects of 2 diets: one based on mild steam cooking (and thus with a low AGEs content: L-AGEs) and another based on high-temperature cooking (H-AGEs). The 2 diets differed mainly in their AGEs content. Authors assessed AGEs in the diet and in subjects' feces, blood, and urine samples, using CML as an indicator of AGEs. In comparison with the L-AGEs diet, 1 month of H-AGEs induced significantly lower insulin sensitivity and plasma concentrations of long-chain n-3 (omega-3) fatty acids and vitamins C and E (−17% (*P* < 0.002), −13% (*P* < 0.0001), and −8% (*P* < 0.01), resp.). However, concentrations of plasma cholesterol and triglycerides increased (+5% (*P* < 0.01) and +9% (*P* < 0.01), resp.). The authors' conclusion was that, in healthy people, a diet based on high-heat-treated foods increases markers associated with increased risk of type 2 diabetes and cardiovascular diseases. These effects can be counteracted by changing the cooking method, that is, by replacing high-heat-treatment techniques by mild cooking techniques.

In contrast to these data, Semba et al. [73] recently published an article showing in 24 healthy subjects that a 6-week H-AGEs diet compared to an isocaloric L-AGEs diet had no impact on endothelial function (measured as peripheral arterial tonometry) and inflammatory mediators. Nevertheless, the conclusion regarding the impact on endothelial function has to be regarded with caution as should be the comparison to our study [18]. The endothelium is the main place where vasoactive humoral factorsare produced accounting for vasodilatation (e.g., nitric oxide, prostacyclin, and endothelium-derived hyperpolarizing factor) or vasoconstriction (e.g., thromboxane A2 and endothelin-1) [77]. Of note, macrovascular function (mirrored by FMD) and microvascular function (mirrored by laser-Doppler measurements) underlies different regulatory mechanisms. While FMD is predominantly mediated by NO [78], the microcirculation is largely independent of NO-mediated regulation and is subject to prostaglandin [79] and non-endothelium-dependent pathways [80]. This explains why a poor correlation exists between the vascular reactivity of the microcirculation and macrocirculation [81]. Moreover, peripheral arterial tonometry and other functional vascular tests are not interchangeable and seem to reflect at least slightly different aspects of endothelial function [82].

## 8. Are the Above-Mentioned Effects due to AGEs?

Dietary AGEs interventions have several drawbacks: (1) blinding of subjects is not possible and therefore studies were performed in the best case investigator-blinded and (2) the findings might have been confounded by the fact that the methods to increase food AGEs content, namely, application of heat, might also affect vitamin activity and the formation of other non-AGEs substances with toxic potential, that is, oxidized lipids.

We therefore performed a first study investigating the effects of a beverage containing small molecules of AGEs.

An AGEs-rich beverage free of carbohydrates or lipids or other known vasoactive substances was administered to diabetic (*n* = 44) as well as healthy subjects (*n* = 10), and its acute effects on arterial endothelial function were assessed by means of FMD measurements as well as measurements of VCAM-1 and plasminogen activator inhibitor-1 (PAI-1) levels. The oral AGEs challenge beverage (300 mL) contained 1.8 × 106 AGE units, but neither carbohydrates nor lipids [71]. The beverage was prepared from glucose and caffeine-free Coca-Cola light, which was concentrated 10 times by rotary evaporation at room temperature. The diabetic subjects had higher baseline levels of serum AGEs (*P* < 0.020), PAI-1 (NS), and VCAM-1 (*P* < 0.033) and lower baseline values of FMD (*P* < 0.032) compared with nondiabetic subjects. Ninety minutes after a single oral AGE challenge, serum AGEs and PAI-1 levels increased and FMD decreased significantly in both healthy subjects (AGEs: 7.2 ± 0.5 to 9.3 ± 1.0 units/mL, *P* < 0.014; PAI-1: 5.4 ± 0.4 to 6.8 ± 0.4 ng/mL, *P* < 0.007; and FMD: 9.9 ± 0.7 to 7.4 ± 0.9%, *P* < 0.019) and diabetic subjects (AGEs: 10.5 ± 0.7 to 14.2 ± 1.0 units/mL, *P* < 0.020; PAI-1: 6.5 ± 1.0 to 10 ± 2 ng/mL, *P* < 0.030; and FMD: 5.4 ± 0.4 to 4.0 ± 0.3%, *P* < 0.032). Serum glucose and VCAM-1 levels remained unchanged. We therefore concluded that significant increases in serum AGEs can occur together with altered clinical measures of endothelial function in diabetic and nondiabetic subjects after a single AGEs-rich beverage.

However, the results have been criticized as potentially not being representative for dietary AGEs derived from common foods, as cola-derived AGEs are only present in low amounts and as small molecules, whereas large AGE modified proteins are missing [83]. It also could not be excluded that substances other than AGEs contained in Coca-Cola might have influenced vascular function. Moreover, the study was not subject-blinded.

We therefore performed a randomized, double-blind, controlled, cross-over study that aimed at investigating the acute effects of dietary AGEs resulting from nonenzymatic glycation during heating of beta-lactoglobulins (a protein class frequently encountered in food) and compared these effects to nonglycated, but heated beta-lactoglobulins (BLG) [84]. Thus, the sole difference between the 2 protein preparations was the advanced protein glycation. Nineteen patients with type 2 diabetes mellitus received on 2 different occasions beverages containing either glycated, heat-treated BLG (AGEs-BLG) or nonglycated, heat-treated BLG (C-BLG). We measured macrovascular (FMD) and microvascular (laser-Doppler measurements of reactive hyperemia in the hand, RH) functions at baseline (*T*_0_), as well as 90 (*T*_90_) and 180 (*T*_180_) minutes after each beverage. Following the AGEs-BLG, FMD decreased at *T*_90_ by 80%^∗‡^ from baseline and remained decreased by 42%^∗^ at *T*_180_ (^∗^ *P* < 0.05 versus baseline, ^‡^ *P* < 0.05 versus C-BLG). In comparison, FMD decrease following C-BLG was lower, with a maximum decrease of 51% at *T*_180_. A significant decrease in nitrite (*T*_180_) and nitrate (*T*_90_ and *T*_180_) as well as a significant increase in CML accompanied the changes following the AGEs-BLG. No change in microvascular function followed any of the 2 beverages.

We concluded that in patients with type 2 diabetes mellitus, an acute oral administration of AGEs from heat-treated, glycated BLG transiently, but significantly impair macrovascular function. These effects are more pronounced than following administration of heat-treated, nonglycated BLG and are accompanied by an increase in circulating CML and a decrease in nitrate. We therefore suggested that the mechanisms leading to vascular dysfunction are related to a decrease in NO bioavailability.

Thus, we demonstrated for the first time in humans that dietary AGEs transiently impair endothelial function in patients with type 2 diabetes mellitus. The relevance for the development of cardiovascular disease still has to be established.

## 9. Conclusions

AGEs play a major role in the development of cardiovascular and diabetes complications as well as other chronic diseases. Dietary AGEs contribute significantly to circulating AGEs and probably to the AGEs pool of the body. Dietary interventions are effective in reducing circulating AGEs in healthy volunteers, patients with diabetes mellitus, and patients with renal failure.

There is compelling data showing that dietary AGEs significantly contribute to the development of postprandial endothelial dysfunction, thus increasing atherosclerotic risk. Accordingly, reducing the dietary AGEs load might represent a cost-effective and easy to implement method showing protective cardiovascular effects. Moreover, a large body of evidence shows that dietary AGEs restriction improves insulin sensitivity and reduces inflammation and oxidative stress. Therefore, beyond recommendations aiming at reducing, for example, dietary fat and carbohydrates, changing the cooking method might also promote health. Uribarri and coworkers [51] published in 2010 a seminal work that reveals the AGEs content of over 540 foods along with recommendations for low-AGEs cooking. They highlighted that dry heat, for example, enhances AGEs formation by 10–100 times compared to the uncooked state across food categories, thus recommending cooking with moist heat, using shorter cooking times, cooking at lower temperatures, and the use of acidic ingredients such as lemon juice or vinegar.

We therefore advocate extending dietary recommendations to healthy cooking. Nevertheless, further studies are needed to better understand the pathogenic effects of food AGEs and to develop strategies for dietary AGEs reduction.
